# Mold contamination in a controlled hospital environment: a 3-year surveillance in southern Italy

**DOI:** 10.1186/s12879-014-0595-z

**Published:** 2014-11-15

**Authors:** Giuseppina Caggiano, Christian Napoli, Caterina Coretti, Grazia Lovero, Giancarlo Scarafile, Osvalda De Giglio, Maria Teresa Montagna

**Affiliations:** Department of Biomedical Science and Human Oncology, Hygiene Section, University of Bari "Aldo Moro", Piazza Giulio Cesare 11, Bari, Italy

**Keywords:** Contamination, Fungi, Hospital, Air, Surfaces, Environment

## Abstract

**Background:**

Environmental monitoring of airborne filamentous fungi is necessary to reduce fungal concentrations in operating theaters and in controlled environments, and to prevent infections. The present study reports results of a surveillance of filamentous fungi carried out on samples from air and surfaces in operating theaters and controlled environments in an Italian university hospital.

**Methods:**

Sampling was performed between January 2010 and December 2012 in 32 operating theaters and five departments with high-risk patients. Indoor air specimens were sampled using a microbiological air sampler; Rodac contact plates were used for surface sampling. Fungal isolates were identified at the level of genera and species.

**Results:**

Sixty-one samples (61/465; 13.1%) were positive for molds, with 18 from controlled environments (18/81; 22.2%) and 43 (43/384; 11.2%) from operating theaters. The highest air fungal load (AFL, colony-forming units per cubic meter [CFU/m^3^]) was recorded in the ophthalmology operating theater, while the pediatric onco-hematology ward had the highest AFL among the wards (47 CFU/m^3^). The most common fungi identified from culture of air specimens were *Aspergillus* spp. (91.8%), *Penicillium* spp., (6%) and *Paecilomyces* spp. (1.5%). During the study period, a statistically significant increase in CFU over time was recorded in air-controlled environments (p = 0.043), while the increase in AFL in operating theaters was not statistically significant (p = 0.145). Molds were found in 29.1% of samples obtained from surfaces. *Aspergillus fumigatus* was the most commonly isolated (68.5%).

**Conclusions:**

Our findings will form the basis for action aimed at improving the air and surface quality of these special wards. The lack of any genetic analysis prevented any correlation of fungal environmental contamination with onset of fungal infection, an analysis that will be undertaken in a prospective study in patients admitted to the same hospital.

**Electronic supplementary material:**

The online version of this article (doi:10.1186/s12879-014-0595-z) contains supplementary material, which is available to authorized users.

## Background

Pathogenic or opportunist microorganisms in hospital environments may be a source of infection in susceptible hosts [1],[2]. This is particularly worrying in controlled environments with immunocompromised patients [3]-[5] and in operating theaters where numerous risk factors may exist, including inefficient ventilation systems and failure to follow infection control behaviors in healthcare workers [6],[7].

The air and surfaces in high-risk environments have been often studied as a source of bacterial infection [8]-[10], but regarding fungal contamination, the literature data are still limited [11]-[14]. The most common infection in fatal complications is aspergillosis in patients with hematological malignancies [15] and mucormycosis in patients with uncontrolled diabetes mellitus [16],[17]. Rare mold diseases, such as fusariosis and scedosporiosis, have also been described [18]-[20]. Filamentous fungal infections (FFIs) are serious postoperative complications in transplant recipients [21],[22], requiring particular attention by the clinician and surgeon.

Although the actual incidence of FFIs has increased worldwide, the real frequency is often underestimated because of the difficulty in determining the cause. Moreover, the lack of standardized protocols and unclear reference threshold values complicates the analysis. The aim of the present study was to analyze the presence of molds in air and on surfaces in operating theaters and controlled environments in a large hospital in southern Italy and to assess the prevalence of the different species that may affect the hospital indoor air quality.

## Methods

### Design study

This study was carried out from January 2010 to December 2012 in five controlled environments (intensive units of wards admitting patients with a high risk of infection) and 32 operating theaters of University Hospital, Apulia, Southern Italy (UHSI). UHSI is a 1400-bed teaching hospital providing all medical services, including a bone marrow and solid organ transplantation service and 32 turbulent air flow operating theaters. According to hospital safety protocols for controlled environments, air and surface microbiological surveillance is performed twice a year in operating theaters and once per year in controlled environments. The 32 operating theaters included eight general surgery, five specialist surgery, four gynecology, four orthopedic, three ophthalmology, three solid-organ transplantation, two otorhinolaryngology, two pediatric, and one maxilla-facial surgery. Controlled environments included a hematology unit (eight rooms), a bone marrow transplant unit (six rooms), a solid-organ transplantation unit (four rooms), a neonatal intensive care unit (NICU) (four rooms), and a pediatric onco-hematology unit (five rooms).

### Air sampling

According to International Standard Organization (ISO 14698-1) [23] microbial standard procedures, air contamination was evaluated by active sampling (SAS, Aquaria Microflow, Milan, Italy) with a flow rate of 180 L/min. Because no air volume is recommended either by the ISO or the Italian Institute for Occupational Safety and Prevention [24], we sampled a suction volume of 500 L of air in one continuous drawing, based on published data [25],[6],[26].

The sampler was placed about 1 m above the floor and 1 m from the operating bed or the patient bed in stay rooms. In each operating theater, sampling was performed twice a year in the early morning before the beginning of surgical activity (*at rest*) to verify the efficiency of environmental cleaning systems and conditioner systems and during surgical activity (*in operational*) to verify the anthropic impact on environmental pollution. In the controlled environments, sampling was performed once a year in the morning after the daily cleaning. A total of 128 samples/year were collected in the 32 operating theaters, of which 64 were *at rest* and 64 *in operational*, and 27 samples/year were collected from the other controlled environments.

The presence of fungi was evaluated using plates containing Sabouraud chloramphenicol dextrose agar (SabC, Becton-Dickinson, Heidelberg, Germany). Each position was simultaneously sampled by two plates that were incubated for 10 days at 28° ±1°C. The number of colony-forming units was adjusted using the conversion table provided by the manufacturer and was expressed in colony-forming units per cubic meter (CFU/m^3^). The results were expressed as the mean of two plates in colony-forming units. The air fungal load (AFL) was defined as the number of captured CFU/m^3^ of air.

### Surface sampling

Surface sampling was carried out using Rodac contact plates contained Sabouraud dextrose agar with chloramphenicol and neutralizing agents (Merck, Grenoble; Becton Dickinson, Rome) according to recommendations of the European Standard - International Organization for Standardization (EN ISO 14698-1) [23]. In controlled environments, one sampling/year was performed at the patient bedhead, wall, air-conditioning unit, night table, and ventilator in all 27 selected rooms. In operating theaters, two samplings/year were performed at the surgical lamp, wall, air-conditioning, and table in all 32 selected rooms. A total of 405 and 768 points were sampled in controlled environments and operating theaters, respectively. Each point was simultaneously sampled by two Rodac plates that were incubated for 10 days at 28° ±1°C. The results were expressed as the mean of two plates in colony-forming units per square centimeter (CFU/cm^2^).

### Fungal identification

Genus and species of the filamentous fungi isolates were identified based on their macroscopic and microscopic morphological features, in accordance with the methods described by de Hoog [27].

The macroscopic examination was based on visual observation of morphological characteristics and color of aerial mycelium, while the microscopic analysis was performed by preparation of lactophenol cotton blue-stained slides. The slides were prepared with tape that adhered to aerial mycelium and placed on the lactophenol cotton blue-stained slides.

### Statistical analysis

Statistical analysis of data was performed using SPSS 10 for Mac OS X (SPSS Inc., Chicago, IL, USA). To assess whether there was a time trend in the fungal contamination, a linear regression with the R^2^ test was fitted to the data.

## Results

### Air

Overall, 465 air samples were collected: 81 from controlled environments and 384 from operating theaters of which 192 were *at rest* and 192 were *in operational*. Sixty-one samples (61/465; 13.1%) were positive for molds, 18 from controlled environments (18/81; 22.2%), and 43 (43/384; 11.2%) from operating theaters. Filamentous fungi were isolated from air samples of two operating theaters (ophthalmology and general surgery) and three controlled environments (solid-organ transplantation unit, neonatal intensive care unit, and the pediatric onco-hematology unit) (Table 1). The highest air fungal load (AFL; CFU/m^3^) was recorded in the ophthalmology operating theater with 61 CFU/m^3^ collected *in operational*, while the highest AFL among the selected wards was in the pediatric onco-hematology (47 CFU/m^3^) (Table 2). Of 402 CFU counted on cultures of all samples, *Aspergillus* spp. was the most frequently recovered (369/402 CFU; 91.8%), followed by *Penicillium* spp., (24/402 CFU; 6%), *Paecilomyces* spp. (6/402 CFU; 1.5%), *Zygomycetes* (2/402 CFU; 0.5%), and *Cladosporium* spp. (1/441 CFU; 0.2%). The annual CFU of *Aspergillus* spp. showed an increase from year 2010 to 2012 (R^2^ = 0.999; p = 0.03) (Table 3, Table 4). A statistically significant increase over time was recorded in controlled environments (R^2^ = 0.996; p = 0.043), while the increase reported in the operating theaters was not statistically significant (R^2^ = 0.949; p = 0.145).

**Table 1:** **Number** (**No**) **of positive air samples in operating theaters** (**OT**) **and controlled environments** (**CE**) **per year**

Point of sampling	No. of positive air samples/year			
	2010	2011	2012	Total
Ophthalmology (OT)	7/12	6/12	12/12	25/36
General surgery (OT)	0/32	8/32	10/32	18/96
Solid organ transplantation (CE)	0/4	2/4	3/4	5/12
NICU^*^ (CE)	2/6	0/6	5/6	7/18
Pediatric onco-hematology (CE)	0/8	2/8	4/8	6/24

*NICU = Neonatal Intensive Care Unit.

**Table 2:** **Fungal isolates recovered in air samples from the operating theaters and controlled environments**

		Total CFU count*/year			
Fungal isolates	2010	2011	2012	No	%
*Aspergillus* spp.	80	125	164	369	91.8
*Penicillium* spp.		3	21	24	6
*Paecilomyces* spp.		6		6	1.5
*Zygomycetes*			2	2	0.5
*Cladosporium* spp.		1		1	0.2
Total	80	135	187	402	100

*The number of CFU counted on cultures of all samples obtained during study.

**Table 3:** **Fungal species recovered in air samples from the controlled environments**

		Total CFU count*/year			
Fungal species	2010	2011	2012	N°	%
*Aspergillus niger*	34	29	52	115	54
*Aspergillus fumigatus*		25	36	61	28.6
*Aspergillus flavus*		10		10	4.7
*Penicillium notatum*		2	15	17	8
*Penicillium expansum*		1	6	7	3.3
*Mucor pusillus*			1	1	0.5
*Rhizopus arrhizus*			1	1	0.5
*Cladosporium herbarum*		1		1	0.5
Total	34	68	111	213	100

*The number of CFU counted on cultures of all samples obtained during study.

**Table 4:** **Fungal species recovered in air samples from the operating theaters**

		Total CFU count*/year			
Fungal species	2010	2011	2012	N	%
*Aspergillus fumigatus*	46	47	51	**144**	**76.2**
*Paecilomyces lilacinus*		6		**6**	**3.2**
*Aspergillus flavus*		14	25	**39**	**20.6**
Total	46	67	76	**189**	**100**

*The number of CFU counted on cultures of all samples obtained during the study.

### Surfaces

In total, 240 surfaces were sampled; 105 samples from 15 operating theatre rooms and 135 from 27 rooms of the controlled environments. Overall, 70 (29.1%) were positive for molds, 48 (68.5%) for *Aspergillus fumigatus*, 14 (20%) for *Aspergillus niger*, and 8 (11.4%) for *Penicillium notatum*. The most contaminated surfaces were the walls and the air conditioning units with a medium of 0.35 CFU/cm^2^ and 0.28 CFU/cm^2^, respectively.

### Invasive fungal infections

During the period study, six cases of invasive fungal infection in two high-risk departments were detected. These cases were defined as *probable* aspergillosis according to the criteria of the European Organization for Research on the Treatment of Cancer/Mycoses Study Group [28]. *Aspergillus niger* in two cases and *Aspergillus fumigatus* in one case, were isolated from the sputum of three patients. In the other three cases of aspergillosis, the diagnosis of *probable* disease was based on clinical and antigen tests (galactomannan assay).

## Discussion

In hospital environments, airborne molds are a potential risk for patients because of possible inhalation of conidia [29]. Because surgical procedures expose patients to infective complications, the operating theater is considered a complex habitat in which all sources of pollution have to be kept under control [21],[22]. In particular, the widespread presence of *Aspergillus* spp. is the major extrinsic risk factor for invasive aspergillosis, caused by *A. fumigatus* and other species of *Aspergillus*, such as *A. flavus*, *A. niger*, and *A. terreus*, depending on the local epidemiology [30] and according to the season [31]. In our study, the AFLs varied from 2 to 47 CFU/m^3^ in the controlled environments and from 0 to 61 CFU/m^3^ in operating theaters, with the highest value reached during surgical procedures. Although we also observed that the AFL varied throughout the year, we could not correlate the data with seasons because of the low number of samples.

The pediatric onco-hematology had the highest fungal contamination, probably because of the presence of more staff and others (parents, clowns to help conventional therapy, and psychologists) or because of the natural migration of fungal spores on the clothes of people coming from outside. This ward underwent partial reconstruction, which raised dust-rich *Aspergillus* spores associated with the construction of nearby buildings, internal demolition, construction, and the renovation of hospital wards. Systems for treating and humidifying air can be easily colonized and create a reservoir of spores in the indoor environment [32].

Studies seem to have confirmed a correlation between *Aspergillus* concentration and cases of aspergillosis. Pini et al. [33], during a surveillance of hematology wards, found extremely low concentrations of *A. fumigatus* in the rooms and corridors and no cases of invasive aspergillosis. More recently, Pokala et al. [14] demonstrated a correlation between a high number of airborne fungal spores and cases of invasive aspergillosis in an onco-hematology pediatric ward that underwent building renovations.

## Conclusions

Although the presence of mold contamination in the healthcare environment may increase fungal infections, the lack of a genetic analysis on recovered strains prevented any correlation analysis between clinical disease and environmental isolates in our surveillance. However, the isolation of fungal strains calls for more adequate control measures. Regular surveillance and cleaning along with restriction of visitors might be among the measures necessary to reduce or totally eliminate the fungal load of indoor air. We are planning a prospective study focused on the correlation of fungal contamination with cases of fungal disease by genetic analysis of clinical and environmental strains, taking into account local epidemiological data.

## Authors' contributions

GC, CN, CC, and MTM contributed to the definition of the study protocol, to the data collection, input, and analysis, and to the manuscript drafting and writing; LG, SG, and DGO contributed to the data collection, input, and analysis. All authors read and approved the final manuscript. GC: Assistant professor, CN: Assistant professor, CC: Graduated student, MTM: Full professor, LG: PHD, SG: Graduated student, DGO: PHD, Department of Biomedical Science and Human Oncology - University "Aldo Moro", Piazza Giulio Cesare 11, Bari, Italy.

## Authors’ original submitted files for images

Below are the links to the authors’ original submitted files for images. Authors’ original file for figure 1 Authors’ original file for figure 2 Authors’ original file for figure 3

## Abbreviations

FFIs: Filamentous fungal infections
UHSI: University Hospital Southern Italy
ISO: International Standard Organization
AFL: Air fungal load
CFU/m^3^: Colony forming units *per* cubic meter
CFU/cm^2^: Colony forming units *per* square centimeter
