# Supplementary figures and images for: Mold contamination in a controlled hospital environment: a 3-year surveillance in southern Italy
Source: BMC Infect Dis. 2014 Nov 15;14:595. doi: 10.1186/s12879-014-0595-z (PMC4236478; doi:10.1186/s12879-014-0595-z)

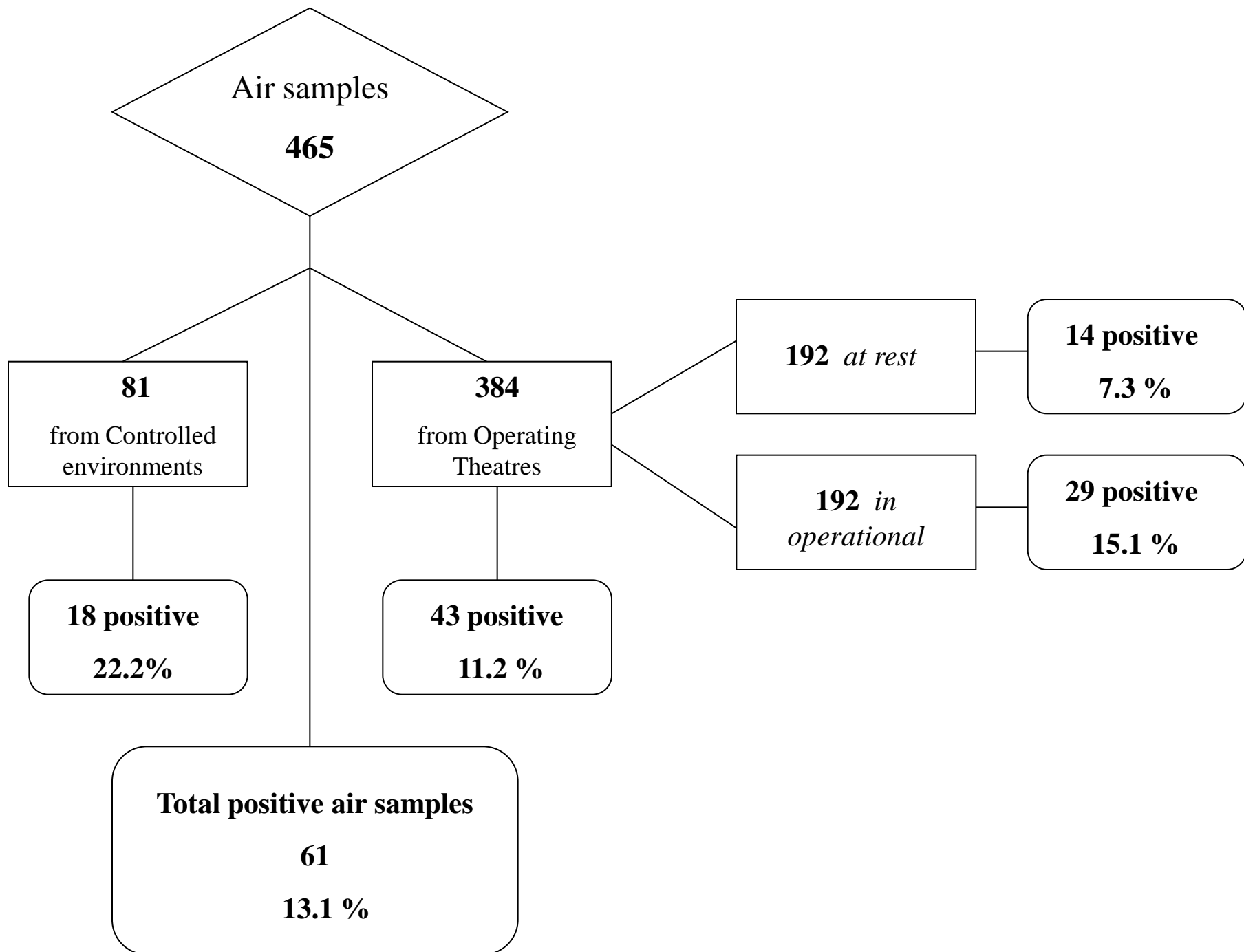

Supplement: Supplementary file 1 — Authors’ original file for figure 1 [file 12879_2014_595_MOESM1_ESM.pdf]
